# The many side jobs of *Lateral suppressor* (*Ls*) in plant development

**DOI:** 10.1093/plphys/kiad496

**Published:** 2023-09-14

**Authors:** Janlo M Robil

**Affiliations:** Assistant Features Editor, Plant Physiology, American Society of Plant Biologists; Department of Biology, School of Science and Engineering, Ateneo de Manila University, Quezon City 1108, Philippines

Shoot architecture in plants is defined by the formation and specification of branches. For millennia, humans have been selecting crop plants based on shoot architecture that favors optimal performance and yield. In recent decades, several genes have been identified to control branch initiation, growth, and determinacy. Among these genes, *Lateral suppressor* (*Ls*), a GRAS transcription factor, affects branching in diverse plants, including tomato, rice, and Arabidopsis (Schumacher et al. 1999; Greb et al. 2003; Li et al. 2003). Mutations in the *Ls* gene and its co-orthologs result in a dramatic reduction in branch or tiller number due to repressed activity of the axillary meristems (AMs). The *Ls* gene is a promising target for crop engineering, but species-specific developmental effects complicate the understanding of its mechanisms (Wang et al. 2018). Therefore, a detailed investigation of the regulatory mechanisms of *Ls* across species and developmental contexts is essential.

In this issue of *Plant Physiology*, Jiang et al. (2023) expand our understanding of *Ls* in commercial watermelon (*Citrullus lanatus*). They found that, in addition to promoting axillary organ formation, *Cl Lateral suppressor* (*ClLs*) also controls tendril and flower identities and leaf morphology (Fig.). Genetic and molecular evidence suggests that sub-functionalization of *ClLs* in watermelon is likely mediated by *ClLs* interacting with different protein partners, supporting a broader involvement of *Ls* in key developmental pathways in plants.

**Figure.**
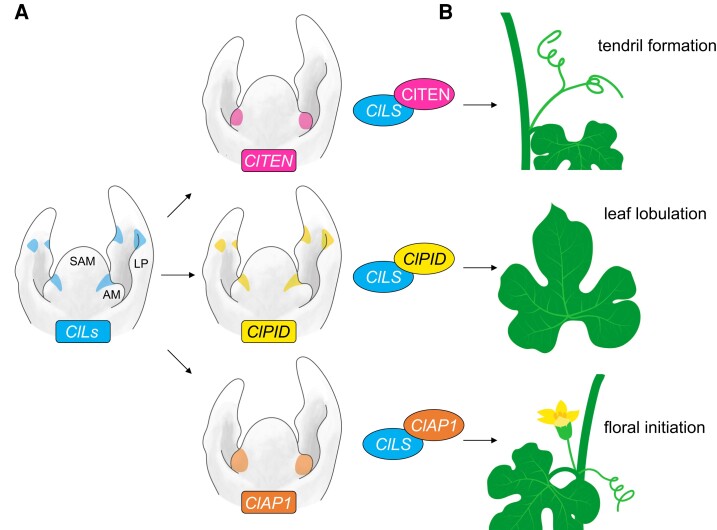
Control of lateral organ patterning in watermelon by *Citrullus lanatus Lateral suppressor* (*ClLs*) through interaction with protein partners. **A)** mRNA expression patterns of *ClLs*, *ClTEN*, *ClPID*, and *ClAP1* in the shoot apex of wild-type watermelon. **B)***Cl*LS protein physically interacts with *Cl*TEN, *Cl*PID, and *Cl*AP1 to regulate tendril formation, leaf lobulation, and floral initiation. AM, axillary meristem; LP, leaf primordium; SAM, shoot apical meristem.

The wild-type watermelon has multiple branches, many of which are modified into tendrils, the threadlike structure that facilitates the plant's clinging habit. The branches, tendrils, and flowers all develop from the axils of the leaves, which are characterized by their lobed leaf blades. In order to determine its role in axillary organ development, the authors generated CRISPR/CAS9-mediated knockouts of *ClLs.* All knockout lines lack branches, tendrils, and flowers, and their leaves appear round due to the absence of lobulation. Those mutant phenotypes support the functional conservation of *Ls* in eudicots but also reveal a previously uncharacterized role of the gene in leaf development.

Jiang et al. (2023) investigated whether the functional expansion in *ClLs* is determined by choosing different protein partners, given that LS has been shown to interact with other proteins to control AM formation (Raman et al. 2008; Rossmann et al. 2015; Guo et al. 2021). First, Jiang et al. (2023) performed a yeast 2-hybrid screen of the wild-type shoot apex and found multiple putative *Cl*LS-interacting partners, including a TCP transcription factor (*Cl*TEN), a serine/threonine kinase (*Cl*PID), and a MADS-box transcription factor (*Cl*AP1). They then conducted a series of in vivo and in vitro assays, all of which show physical interaction of *Cl*LS with those 3 proteins. The new interacting partners for *Cl*LS could potentially help explain its sub-functionalization in watermelon.

In order to test whether there are overlapping gene activities between *ClLs* and *ClTEN, ClPID,* and *ClAP1*, the authors investigated their mRNA expression patterns in the developing shoot apices by in situ hybridization. As expected, *ClLs* is expressed in the axils of the AMs and leaf primordia. However, in the leaf primordia, *ClLs* is also expressed in narrow regions of the future leaf blade. Of the 3 interacting genes, only *ClPID* shares similar expression domains with *ClLs*. In contrast, both *ClTEN* and *ClAP1* are exclusively expressed in the AMs that correspond to incipient tendril primordia and floral meristems, respectively. Those observations suggest that *ClLs* and *ClPID* have overlapping gene activities in the axils and developing leaves. On the other hand, the expression of *ClTEN* and *ClAP1* in AMs, where *ClLs* is excluded, suggests antagonistic gene activities and raises interesting questions about their regulatory mechanisms.

Finally, to further investigate the functions of *ClTEN, ClPID*, and *ClAP1*, Jiang et al. (2023) generated knockout lines of each gene with CRISPR/Cas9. In *Clten* mutants, tendrils are either absent or replaced by branches that exhibit tendril-like curvatures. *Clpid* mutants exhibit a dwarf and compact stature, produce flowers with missing lateral organs, and have notably rounder leaves that lack lobes. Although *Clap1* mutants exhibit normal vegetative structures, they completely fail to produce flowers throughout their development. In addition, the authors found that the transcript levels of *ClLs* were significantly reduced in all those knockout lines. Altogether, *Clten*, *Clpid*, and *Clap1* mutants recapitulate distinct phenotypes observed in *ClLs* mutants, providing further evidence for overlapping functions of those 3 genes with *ClLs* in the patterning lateral organs in watermelon.

The authors conclude that *ClLs* had undergone sub-functionalization in watermelon, with *Cl*LS protein interacting with *Cl*TEN, *Cl*PID, and *Cl*AP1 proteins to control the development of tendrils, leaves, and flowers, respectively (Fig.). However, further analysis is needed to understand the specific mechanisms of those interactions. For example, it will be important to determine whether *Cl*PID phosphorylates *Cl*LS. Additionally, since the expression domains of *ClTEN* and *ClAP1* do not overlap with those of *ClLs*, it will be important to investigate whether these proteins interact with *ClLs* through non-cell autonomous signaling. Moreover, future studies should clarify the roles of auxin, gibberellic acid, and strigolactones in *ClLs* sub-functionalization, based on the known relationships of its interacting proteins with these hormones (Wang et al. 2018).

The work of Jiang et al. (2023) has implications for crop breeding as it highlights the challenges and limitations of targeting a single gene to modify plant architecture. The long-term goal of the study was to improve watermelon fruit harvesting efficiency by reducing multiple branching, but the unintended effects of disabling *ClLs* on the development of other vegetative and reproductive organs underscore the complexity of the precise mechanisms controlling axillary organ formation. Yet, the study reveals new and important information about the diverse functions of *Ls,* which could pave the way for improving shoot architecture in watermelon and other crop species.

## Dive Curated Terms

The following phenotypic, genotypic, and functional terms are of significance to the work described in this paper:

am AmiGo: PO:0020144
